# *Saccharomyces cerevisiae* as a Model Organism: A Comparative Study

**DOI:** 10.1371/journal.pone.0016015

**Published:** 2011-02-02

**Authors:** Hiren Karathia, Ester Vilaprinyo, Albert Sorribas, Rui Alves

**Affiliations:** 1 Departament Ciències Mèdiques Bàsiques, Universitat de Lleida & IRBLleida, Lleida, Spain; 2 Evaluation and Clinical Epidemiology Department, Hospital del Mar-IMIM, Barcelona, Spain; Cajal Institute, Consejo Superior de Investigaciones Científicas, Spain

## Abstract

**Background:**

Model organisms are used for research because they provide a framework on which to develop and optimize methods that facilitate and standardize analysis. Such organisms should be representative of the living beings for which they are to serve as proxy. However, in practice, a model organism is often selected *ad hoc*, and without considering its representativeness, because a systematic and rational method to include this consideration in the selection process is still lacking.

**Methodology/Principal Findings:**

In this work we propose such a method and apply it in a pilot study of strengths and limitations of *Saccharomyces cerevisiae* as a model organism. The method relies on the functional classification of proteins into different biological pathways and processes and on full proteome comparisons between the putative model organism and other organisms for which we would like to extrapolate results. Here we compare *S. cerevisiae* to 704 other organisms from various phyla. For each organism, our results identify the pathways and processes for which *S. cerevisiae* is predicted to be a good model to extrapolate from. We find that animals in general and *Homo sapiens* in particular are some of the non-fungal organisms for which *S. cerevisiae* is likely to be a good model in which to study a significant fraction of common biological processes. We validate our approach by correctly predicting which organisms are phenotypically more distant from *S. cerevisiae* with respect to several different biological processes.

**Conclusions/Significance:**

The method we propose could be used to choose appropriate substitute model organisms for the study of biological processes in other species that are harder to study. For example, one could identify appropriate models to study either pathologies in humans or specific biological processes in species with a long development time, such as plants.

## Introduction

The use of model organisms for research is a hallmark of scientific endeavor (e.g. [1], [2], [3], [4], [5], [6], [7]). Such organisms are used because a) they may help overcomes ethical and experimental constraints that hold for the target life form, b) they provide a framework on which to develop and optimize analytical methods that facilitate and standardize analysis, and c) they are thought to be representative of a larger class of living beings for whatever biological phenomenon or process the community is interested in. However, the choice of a model organism is often guided more by the first two considerations than by the last one. Nevertheless, selection of a model organism based on accumulated technical experience and on availability of experimental techniques does not guarantee representative results in other organisms. In fact, a gap exists in systematically establishing how close different organisms are with respect to a given process, before choosing one of them as a model for studying that process.

Such a choice should be informed by several considerations. First, the processes of interest for comparison must be clearly identified. Then, one should establish a qualitative or quantitative metric that measures similarity between the different organisms with respect to those processes. Finally, the processes of interest should be sufficiently well characterized in the alternative organisms so that the metric can be used for comparison. If rigorously performed, this final step defeats the purpose of using the model system as a tool to extrapolate from, because all organism would be rigorously characterized beforehand. In fact, this characterization (by proxy) is the purpose of using a model organism. Therefore, methods that rationally predict how similar different organisms might be with respect to biological processes of interest are needed.

The accumulation of fully sequenced genomes [8] and the advances in comparative genomics [9], [10] and computational systems biology [11] allows us to develop such methods. This can be done by applying strategies that compare the protein or gene networks involved in the process of interest in order to establish a similarity ranking that can be used to predict, to a first approximation, the accuracy of extrapolating the behavior of specific processes between organisms. Testing this idea requires a thorough analysis of the molecular circuits in a well-known model organism and a comparison of these circuits to those in other living beings.

To do this we have choose the yeast *Saccharomyces cerevisiae* (*S. cerevisiae*) to perform a pilot study. This yeast is one of the most widely used eukaryotic model organisms. It has been used as a model to study aging [12], regulation of gene expression [13], signal transduction [14], cell cycle [15], metabolism [16], [17], apoptosis [18], neurodegenerative disorders [19], and many other biological processes. For example, up to **30%** of genes implicated in human disease may have orthologs in the yeast proteome [20].

We use the protein networks that are involved in specific biological processes to compare the differences between *S. cerevisiae* and 704 other organisms, and predict in which organisms the different processes should behave more similarly to the corresponding process in the yeast. We validate some of the predictions by comparing the dynamic behavior of a number of specific pathways in different organisms to that of the corresponding pathway in *S. cerevisiae*.

Our results suggest that the method proposed here is adequate for its purpose. Furthermore, they support the use of *S. cerevisiae* as a model organism to study different processes, while pinpointing specific biological phenomena from this yeast that may not be readily comparable to their analogous processes in other organisms. The method we propose here could be especially relevant to assist in the choice of appropriate model organisms for both, the study of human specific biological processes and the characterization of a specific biological phenomenon in a large class of organisms. It could also be useful in choosing appropriate models for processes in organisms, such as plants, that due to their long duplication times cannot be easily studied.

## Results

### Strategy for the comparison of different processes in different organisms

The strategy we use to establish how similar a given process is in two different organisms is as follows. First, we identify orthologs (i.e. genes in different species that evolved from a common ancestral gene by speciation) between the genome of the potential model organism and that of the target organism(s). Then, we attribute function to the different genes in the organisms under comparison and assign each gene to specific biological processes, using biological ontologies [21]. Specifically, we use:

- The Gene Ontology (GO) [22], which has been widely used for annotating function and localization of genes at a coarse level in many organisms [23], [24], [25], [26], [27], and
- The pathways that regulate and execute the processes that one is interested in studying, as defined in KEGG [28] (one can also use MetaCYC [29]).

Finally, we compare the sets of genes responsible for the different processes that are present in each organism. Such an approach predicts if two organisms are likely to be comparable with respect to specific processes of interest, by establishing whether the elements that are a part of the molecular circuits executing the relevant processes are analogous between the organisms (see methods for further details).

Because this is a pilot study, we focus on an organism that is widely used and well characterized, *S. cerevisiae*. We have attributed function to each of the proteins in *S. cerevisiae*, according to the information derived from GO and KEGG. This allowed us to create a functional classification of the proteins with respect to the biological processes that they are involved in. Details about this classification are given in Figure S1 and Tables S1–S3 materials. With the functional classification of proteins in place, we can compare the different molecular circuits and processes of yeast to their analogs in 704 other organisms.

To compare these molecular circuits and biological processes between *S. cerevisiae* and other organisms, we created clusters of **orthologs** (ScCOGs: *S. cerevisiae* Clusters of Orthologs), **homologues** (ScCHGs: *S. cerevisiae* Clusters of Homologues) and **absent** proteins (ScCAGs: *S. cerevisiae* Clusters of Absent Genes) for each *S. cerevisiae* protein with respect to the translated genome of each of the other 704 organisms. Hereafter we only discuss the results for ScCOGs, because these are consistent with those for ScCHGs. The results for each organism are summarized in Table S1. The detailed clusters are provided as Text S1 and Text S2. We are also preparing a server where these results can be further explored and the method can be applied to other organisms.

Each cluster was associated with the functional terms corresponding to its *S. cerevisiae* protein. To analyze the differences between *S. cerevisiae* and a specific organism with respect to a given process, we compare the fraction of proteins that are annotated as functioning in that process in both organisms. We investigate if orthologs or homologues for each of these proteins are simultaneously present in both organisms or not. Then, we rank organisms with respect to the differences in the set of proteins responsible for each process, analyzing for ScCOGs, ScCHGs and ScCAGs at the level of domain, kingdom and phyla for all the 704 organisms (summarized in Tables S2 and S3).

### Functional comparison of the full *S. cerevisiae* protein complement to that of archaea, bacteria and eukaryotes

We compared how well the proteins in the different ScCOGs, ScCHGs and ScCAGs are conserved between *S. cerevisiae* and various classes of organisms. This allowed us to predict if *S. cerevisiae* can be a good model for specific processes in different classes of organisms, rather than in individual species. The details of the analysis are presented in appendix S1. No *S. cerevisiae* protein has orthologs in all 704 organisms. Furthermore, no *S. cerevisiae* protein has homologues in all the Prokaryotes (Archaea & Bacteria together). In addition, 2642 (45%) *S. cerevisiae* proteins are absent in all the Prokaryotes (for more details see Tables S2 and S3).

### ARCHAEA DOMAIN

We analyzed 48 species of *Archaea*. About 20% (1158) of all *S. cerevisiae* proteins generate ScCOGs that contain *Archaea* sequences. However, only 2% (103) of all yeast proteins generate ScCOGs that contain at least a sequence from each sequenced species of *Archaea*. An additional 18 (0.3%) *S. cerevisiae* proteins have homologues in all *Archaea*. 3672 (62%) *S. cerevisiae* proteins are absent in all *Archaea*. Most of these have unknown function. Overall, there is no group of organisms for which the networks of proteins responsible for a large fraction of biological processes in *S. cerevisiae* are similar to their counterparts in *Archaea*. However, some biological processes are predicted to be similar between *S. cerevisae* and some *Archaea* (see below).

### BACTERIA DOMAIN

We analyzed 598 species of *Bacteria*. 1612 (27%) of all *S. cerevisiae* proteins generate ScCOGs that contain bacteria sequences. However, no ScCOG or ScCHG contains a sequence from each bacterial species. Furthermore, 2881 (49%) *S. cerevisiae* genes are absent from all *Bacteria*, a smaller percentage than that for *Archaea*. As was the case in archaea, overall, there is no group of organisms for which the networks of proteins responsible for a large fraction of biological processes in *S. cerevisiae* are similar to their counterparts in *Bacteria*. However, some biological processes are predicted to be similar between *S. cerevisae* and some *Bacteria* (see below).

### EUKARYOTA DOMAIN

Overall, there are 59 species of *Eukaryotes* in our dataset. About 4.5% (263) of all ScCOGs contain sequences from each of these organisms. Between 40% and 60% of all *S. cerevisiae* proteins involved in “MAPK signaling pathways”, “Signal transduction” biological process, and “Helicase activity” molecular functions have orthologs in all 59 species. Furthermore, between 60% and 80% of all proteins involved in “Microtubule organizing center” of *S. cerevisiae* are also found in all 59 sequenced eukaryotes. Overall, the networks of proteins responsible for a large fraction of biological processes in *S. cerevisiae* are similar to their counterparts in *ascomycetes*. Furthermore, several biological processes are predicted to be similar between *S. cerevisae* and other *Eukaryotes*.

A more detailed analysis of the three domains is given in Appendix S1.

### Functional comparison of biological processes and pathways between *S. cerevisiae* and other organisms

After getting such a bird's eye view of the similarities and differences between *S. cerevisiae* and different clades of organisms with respect to different biological processes, we now focus on individual organisms. To obtain an approximate estimation of how close a given biological process is between *S. cerevisiae* and another organism we build a matrix of 704×5880 entries. In this matrix, a row represents an organism, while a column represents a ScCOG. The matrix entries are 0 if no sequence from the corresponding organism is found in the appropriate ScCOG and 1 otherwise.

Then, we build a secondary set of four additional matrices containing information about KEGG pathways, biological processes, molecular activity and cellular localization. In each matrix, the rows represent the organisms and the columns represent the biological process, the cellular localization, the molecular function, or the KEGG pathway. Each entry in one of these matrices is a vector with a variable number of elements that is constant for each column of a matrix. The number of elements in the vector is equal to the number of different proteins that is associated to the specific biological process or pathway corresponding to the column (See methods for details).

Subsequently, we calculate the Normalized Hamming Distance (NHD) between the vector of proteins in one entry of the matrix and the corresponding vector for *S. cerevisiae* from that same column. This NHD is a metric based on the number of elements that are different between the two vectors. The smaller the NHD, the more similar the two vectors are and the more similar the set of proteins executing a specific process in both organisms is. Consequently, the more likely it is that *S. cerevisiae* is a good model to study the relevant process and generalize the results to the other organism. Using this metric we have clustered the organisms in the matrix according to growing overall NHD with respect to *S. cerevisiae*.

### KEGG Pathways

**Figure 1:**
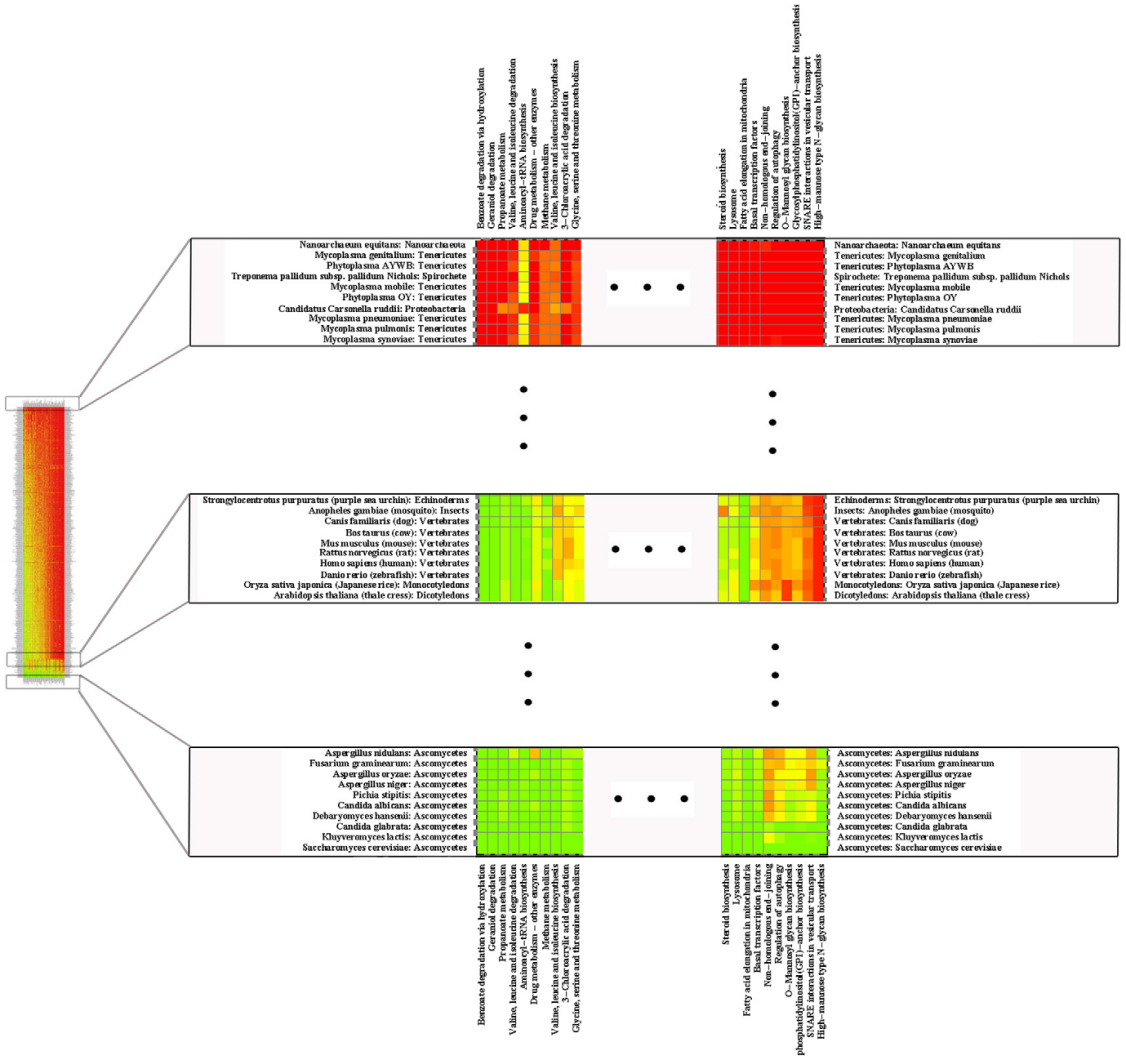
Details of a heat-map representation showing how distant each organisms is from *S. cerevisiae* with respect to each individual KEGG pathway. A green square indicates a high level of coincidence between the set of proteins involved in the specific pathway (column) in a given organism (row) and the set of proteins for the same pathway in *S. cerevisiae*. A red square indicates complete absence of the set of proteins involved in the specific pathway (column) in a given organism (row) with respect to the same pathway in *S. cerevisiae*. Intermediate colors indicate intermediate degrees of coincidence between the set of proteins in the target organism and that in *S. cerevisiae*. The complete heat-map can be seen in Figure S1.

Figure 1 summarizes the results for KEGG pathways (see Figure S2 for a complete analysis). “Benzoate degradation via hydroxylation”, “Geraniol degradation”, “Propanoate metabolism”, “Valine, leucine and isoleucine biosynthesis”, “Glycolysis/Gluconeogenesis”, “methane metabolism”, “Glycolysis/Gluconeogenesis” and “Aminoacyl-t-RNA biosynthesis” are pathways that appear to be similar to those of *S. cerevisiae* in a large fraction of organisms. Pathways such as *S. cerevisiae*'s “RNA polymerase” (29 genes), “Lysosome” (14 genes), “Endocytosis” (33 genes), “Oxidative phosphorylation” (76 genes), “Ribosome” (142 genes), “MAPK signaling pathway - yeast” (55 genes), “DNA replication” (30 genes), and “Ubiquitin mediated proteolysis” (44 genes) and “Nucleotide excision repair” (34 genes) are much more similar to those from other eukaryotes than to the corresponding prokaryotic pathways (when they exist). Among the pathways that are central for life, the one that appears to be more unique to *S. cerevisiae* and other Saccharomycetes is cell cycle (115 genes), because only a small fraction of its proteins have orthologs in other eukaryotes. Thus, these results suggest that extrapolating cell cycle studies in *S. cerevisiae* to other organisms outside of the Saccharomycetes clade should be done only at the level of basic principles, if at all (see for example [30], [31]). A more detailed analysis of these pathways and their similarity between *S. cerevisiae* and the other 704 organisms can be found in the appendix and in Figure S2.

An encouraging observation for the use of *S. cerevisiae* as a model organism for mammals is that most of the studied mammals (humans, dogs, mice, cows and rats) are among the non-fungal organisms that have biological processes with protein sets that are similar to the corresponding sets of *S. cerevisiae*. Specifically, the sets of *S. cerevisiae* proteins that are associated to “Mismatch repair” (18 genes), “Ubiquitin and other terpenoid-quinone biosynthesis” (5 genes), “Inositol phosphate metabolism” (15 genes), “Steroid biosynthesis” (15 genes), “Ubiquitin mediated proteolysis” (44 genes), “DNA replication” (30 genes), “Ribosome” (142 genes), “Proteasome” (35 genes), “Mismatch repair” (18 genes), “Galactose metabolism” (23 genes), “One carbon pool by folate” (14 genes) and “Glycolysis/gluconeogenesis” (48 genes) are those that appear to be more similar to the corresponding sets of proteins in man. A more thorough analysis is given in the Appendix S1.

### GO Biological Processes, Cellular Component and Molecular Function

**Figure 2:**
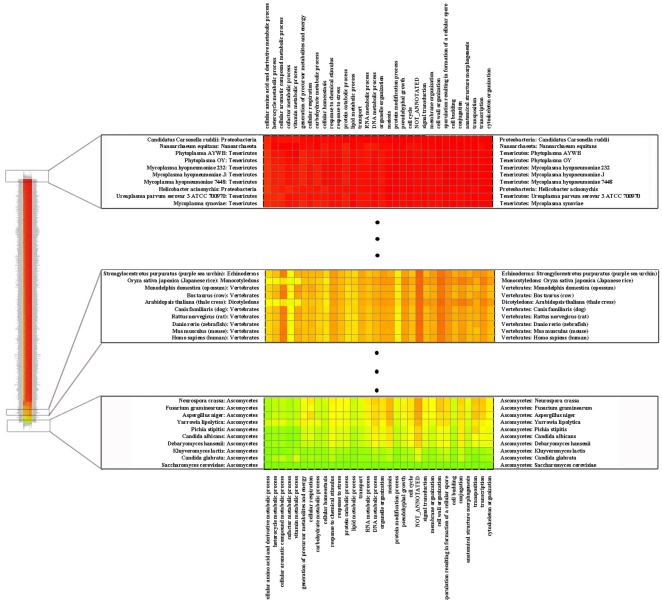
Details of a heat-map representation showing how distant each organisms is from *S. cerevisiae* with respect to each biological process from the GOSLIM classification. A green square indicates a high level of coincidence between the set of proteins involved in the specific biological process (column) in a given organism (row) and the set of proteins for the same pathway in *S. cerevisiae*. A red square indicates complete absence of the set of proteins involved in the specific pathway (column) in a given organism (row) with respect to the same biological process in *S. cerevisiae*. Intermediate colors indicate intermediate degrees of coincidence between the set of proteins in the target organism and that in *S. cerevisiae*. The complete heat-map can be seen in Figure S2.

**Figure 3:**
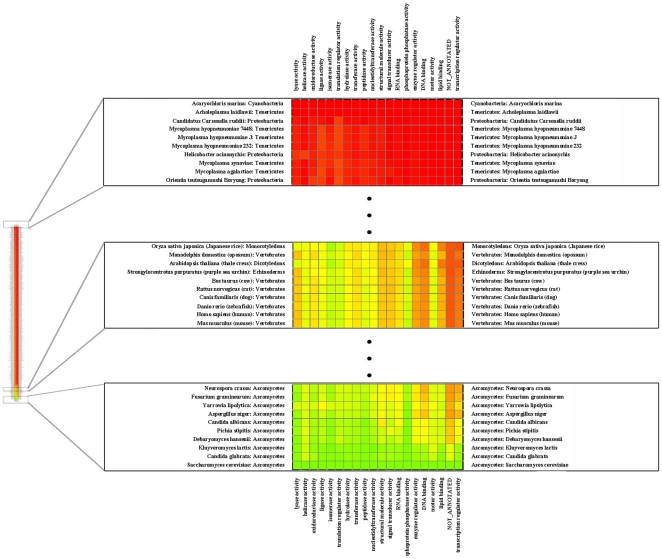
Details of a heat-map representation showing how distant each organisms is from *S. cerevisiae* with respect to each molecular function from the GOSLIM classification. A green square indicates a high level of coincidence between the set of proteins involved in the specific molecular function (column) in a given organism (row) and the set of proteins for the same pathway in *S. cerevisiae*. A red square indicates complete absence of the set of proteins involved in the specific pathway (column) in a given organism (row) with respect to the same molecular function in *S. cerevisiae*. Intermediate colors indicate intermediate degrees of coincidence between the set of proteins in the target organism and that in *S. cerevisiae*. The complete heat-map can be seen in Figure S3.

Figures 2 and 3 summarize the results for the comparisons between *S. cerevisiae* and the other organisms using the GO categories classification. Details can be further analyzed in Figures S3, S4 and S5. The results are similar to those described for Figure 1 (or those reported in Figure S2), which suggests that these functional classifications are, to a large extent, equivalent, in spite of all problems that they might have (see discussion). *S. cerevisiae* metabolic activities like “Cellular amino acid and derivative metabolic process”, “Cellular aromatic compound metabolic process”, “Heterocycle metabolic process”, “Cofactor metabolic process” and “Vitamin metabolic process” are the ones that are more conserved in all organisms. In contrast, “cytoskeleton organization”, “Transcription”, “Anatomical structure morphogenesis”, “Transposition”, “conjugation”, “Cell budding”, and “Protein modification process” appear to be conserved mostly in eukaryotes. Conservation of the “Cell wall organization” pathway is restricted to fungi.

### Validating the predictions

The analysis described above and the results given in Figures 1, 2 and 3 and in Figures S2-S5 ranks the difference between the protein set responsible for a given biological process in each organism and the corresponding set in *S. cerevisiae*. If our earlier arguments are correct, one would expect that the similarity between the adaptive responses that involve a given process in other organisms and the same responses in *S. cerevisiae* is directly correlated to the similarity between the protein sets that regulate and execute that process.

In other words, we define a static metric of closeness of processes between organisms that is based solely on the similarity between the sets of proteins involved in those processes in both organisms. Can we assume that such a metric is also a good measure of closeness between physiological and adaptive responses of the pathways regulating the processes in the organisms being compared, even though it does not include any kinetic or regulatory information?

To answer this question we selected pathways for which dynamic, regulatory, and/or phenotypic information was available for *S. cerevisiae* and for a scope of different organisms. This selection was based on a careful analysis of Figure S3. We systematically identified pathways or processes with more than 4 genes and then searched the literature for comparable studies of the dynamical and adaptive behavior of these processes in different organisms that belong to our dataset. We were able to identify twelve cases that could be used to answer the question from the previous paragraph.

The results are summarized in Table S4. They show that the phenotypic adaptations and dynamical behavior of a given pathway is more similar to that of *S. cerevisiae* in organisms that are found to be closer to *S. cerevisiae* according to our analysis than in more distant organisms. Thus, even if the method we propose is based on static information, the results of the analysis appear to be adequate for pinpointing an appropriate model organism from which to study and extrapolate the dynamical and adaptive behavior of specific biological processes.

## Discussion

### The rational choice of model organisms and its technical limitations

In this work we ask the question “How can one chose an appropriate model organism in which to study a specific biological process in such a way that the results may be extrapolated to another organism?” We propose a systematic way to answer this question that involves comparing the similarity between the set of proteins that participate in the biological process of interest in the organism to the equivalent set of proteins in the organism to which we want to extrapolate the results. The closer the set of proteins is between the two, the more likely it is that the results from one organism can be extrapolated to the other.To compare the sets of proteins between organisms, we propose a procedure that involves: a) associating a protein to a process or pathway, for example using GO categories or the KEGG pathways, and b) compare the sets of proteins associated to the process between the relevant organisms. This method offers a proxy for establishing probable equivalency of processes between organisms, but it has some drawbacks.

First, more often than not, there will be little functional information associated to the proteins of a given organism. To overcome such a problem, we propose choosing an initial subject organism that is well studied and functionally well characterized at the molecular level. As our method relies on ortholog identification and functional annotation, it requires that this annotation be continuously improved even in well studied organisms. By choosing *S. cerevisiae* as an example we use the eukaryotic organism that we believe has the best overall functional annotation. It must also be emphasized that, when comparing the set of proteins that participate in a given process in different organisms, one must consider the “super set” of proteins participating in that process and compare the differences. In other words, for example when comparing KEGG pathways, one can consider the pathway that includes all possible EC numbers and then compare the two organisms in this context. This was also done here. Otherwise, one may find a situation where two organisms are predicted as being good models with respect to a given process when the proteins in one organism are a small subset of those in the other.

Second, using sequence similarity to establish functional orthology also has its drawbacks. On one hand, sometimes functional orthology exists even in the absence of sequence orthology and vice versa. Comparing the structures of proteins as well as their amino acid motifs and active centers provides some assistance in tackling this problem. However, at the current stage of development in bioinformatics, sequence comparison is still the most efficient and accurate way to make such predictions on the scale that we made them for this work. On the other hand, sometimes, due to gene duplication and domain shuffling, proteins that are unique in one organism may have several close sequence homologues in another. We address this problem by proposing a procedure that takes several similarity factors between sequences into account before deciding which of the homologues is the more likely to be orthologous to the query protein. These factors include e-value score, similarity of the sequences and the fraction of the two proteins that is comparable. Nevertheless, if one also analyzes homologues separately, as we also do here, one stands a better chance of controlling for false negative orthologs.

Third, by comparing only the set of proteins associated with a given biological process in different organisms, we are disregarding regulatory and dynamic information that could be important for the comparison. This shortcoming may not be problematic. On one hand our method is a good way to eliminate processes and organisms for which the reference organism is not a good model. If the sets of proteins that execute a given process are very dissimilar, then the dynamics are not even an issue because other model organisms need to be chosen. On the other hand, having a more similar set of proteins associated to a specific process makes it more likely that the adaptive and regulatory responses of the process be similar. This claim can be supported by comparing the physiological responses of different organisms to that of the model organism (see below).

Fourth, sometimes the logic used to define the proteins associated to specific biological pathways or processes is questionable. This is a very important factor and a successful general application of the method described here requires that the annotation of genomes and ontologies/pathways keeps on improving. Poorly characterized biological processes will lead to greater errors in the comparisons. There is little we can do with respect to this limitation at this time. One of the actions that can be taken to minimize this problem is to choose as a model an organism that is one of the best annotated worldwide. We did so by choosing *S. cerevisiae* as a model for the study. This organism has the additional advantages of being well characterized at the molecular level and used to study many biological processes that are important in other organisms. To further ameliorate this problem we carefully curated both the KEGG and GO associations of yeast.

### *S. cerevisiae* as a model organism

We apply our method to a pilot study of *S. cerevisiae* as a model organism, by comparing it to 704 other organisms. The results are presented in detail in Figures 1, 2 and 3, Figures S1–S5 and Tables S1–S4. In *S. cerevisiae* 4571 proteins are not associated to any pathway in the KEGG database. Analyzing the approximately 1000 proteins that have such a functional association, we find that, as expected, in many cases evolutionary closeness goes on par with similarity between sets of proteins that are associated to a specific biological process.

As mentioned above, our inference of closeness between *S. cerevisiae* and the other organisms is based upon an analysis of similarity between the sets of proteins involved in a specific process in both organisms. This analysis does not include any information about the physiological responses and the dynamic or regulatory aspects of the biological processes and pathways being compared between organisms. To understand if this limitation is in general important we selected pathways for which dynamic, regulatory, and phenotypic information was available for *S. cerevisiae* and for a scope of other different organisms. We then compare the behavior of those pathways in yeast and in the other organisms. In this comparison, organisms that are predicted to be closer for a specific pathway or process also have more similar adaptive responses (Table S4). Furthermore, recent work that uses orthology between human genes and those in other organisms to find models for human diseases support these results [32], [33], [34]. Together, this suggests that our method is adequate both for eliminating unsuitable model organisms and for choosing an appropriate model organism from which to study and extrapolate the dynamical and adaptive behavior of specific biological processes.

### Conclusion

Our results support the use of *S. cerevisiae* as a model organism to study different biological processes and pathways in specific organisms, while pinpointing specific processes in this yeast that may not be readily generalizable to other organisms. We conclude that using a single proteome as a reference and applying a methodology such as the one suggested here, one can in general appropriately select model organisms to study the dynamic and adaptive responses of a given biological process, as long as the proteins that participate in that process are known.

## Materials and Methods

### Selection of genome sequences

The complete proteome of *Saccharomyces cerevisiae* (5880 proteins) was downloaded from NCBI (December 2009). The complete sequences for the full protein complement of 704 organisms with fully sequenced genomes was downloaded from the KEGG database (December 2009) and cross-referenced to that provided the NCBI database.

### Homology analysis

We downloaded BLAST version 2.2.18 from NCBI and used it locally. All genome and protein sequences were formatted using FormatDB. A pipeline for selecting orthologous proteins, homologous proteins and proteins of the *S. cerevisiae* that are absent in each of the other organisms was developed and implemented in PERL.

### Orthology analysis

The collection of all proteins in a target genome that blasted against a specific protein of *S. cerevisiae* with an e-value ≤10^−10^ was analyzed. Manually and through the comparison of the *S. cerevisiae* proteome to that of two organisms from each class, we setup a cutoff value for separating orthologs from homologues. Pairs of proteins with e-value between 10^−10^ and 10^−36^ and identity score below 30% are considered as homologues. If the alignment spans over 85% of either sequence and either the e-value of the blast search is bellow 10^−36^ or the identity score is higher than 30%, both proteins are considered as belonging to the same family of orthologs [35]. When more than one protein in a target genome meets these conditions with respect to the same *S. cerevisiae* protein we calculate an orthology score function, **F**. The protein with the highest **F**-score function is considered to be the most likely ortholog with respect to the *S. cerevisiae* protein, while the remaining proteins are flagged as in-paralogs of that ortholog. **F** is defined as follows:
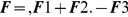
(Eq.1)


Factor **F1** is calculated as follows.
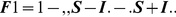
(Eq.2)


In Eq. 2, **S** represents the similarity score and **I** represent the identity score of the alignment. **F1** is always between 0 and 1. The more similar two sequences are, the closer to 1 will F1 be.

Factor **F2** is calculated as follows.
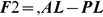
(Eq.3)


In Eq. 3, **AL** represents the length of the alignment, and **PL** is the total length of the query sequence. F2 is always between 0 and 1. The larger the fraction of the query sequence that aligns with the target sequence is, the more similar the two proteins will be and the closer to 1 will F2 be.

Finally, factor **F3** is calculated as follows.
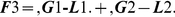
(Eq.4)


In Eq. 4, **G1** represents the number of gaps within the aligned region of the query sequence, **L1** represents the length of the query sequence, **G2** represents the number of gaps within the aligned region of the target sequence, and **L2** represents the full length of the target sequence. The closer to zero **F3** is the more similar will the two sequences be.

Theoretically, -∞≤**F**≤2. However, in practice, we found that **F** typically assumes values between 0 and 2. The higher **F** is, the more likely it is that the query and target sequence are orthologs.

**Figure 4:**
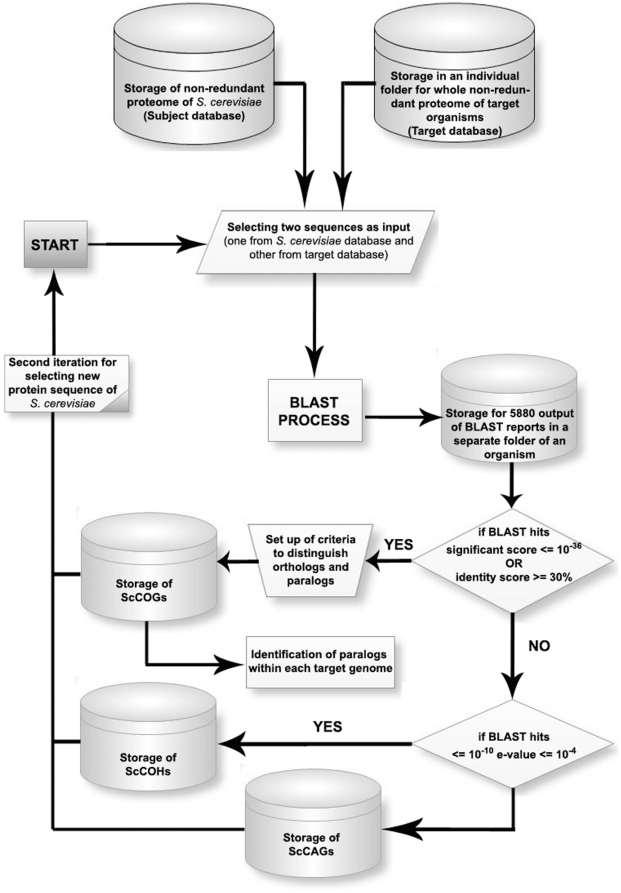
Summary of the process used to build ScCOGs, ScCHGs and ScCAGs. The full proteome of *S. cerevisiae* was compared to the full proteome of each of 704 different organisms using BLAST. See methods for details.

The whole process is summarized in Figure 4. At the end of the analysis we obtain clusters of **orthologs** (ScCOGs) and **homologues** (ScCHGs) for all the *S. cerevisiae* genes with respect to the other 704 organisms. We also obtain a third family of clusters (ScCAGs), that of proteins from *S. cerevisiae* that are absent **from** the target genomes.

### Classification of clusters according to pathways and biological processes

In order to attribute biological function to the ScCOGs, ScCHGs and ScCAGs, we implemented the following procedure. On one hand, we used the GOSLIM classification of gene function for *S. cerevisiae* from SGD [36], [37] to attribute biological function, molecular functions and cellular localization to each cluster. On the other, we downloaded data from KEGG that associates genes to KEGG metabolic circuits in fully sequenced genomes [38] and attribute pathways terms to each of the clusters.

### Calculation of the Hamming distance

The Hamming Distance (HD) between the vector,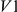. of protein functions associated to a specific process, localization or pathway in *S. cerevisiae* and the vector, 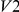. of corresponding protein functions in another organism gives a measure of how different the two vectors are. It is calculated using the formula 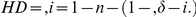. where,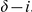 is the Kronecker delta, 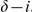 is 1 if the elements in position *i* of both vectors are orthologs and 0 otherwise. The smaller the distance, the more similar the two vectors are and the more similar is the set of genes executing a specific process in both organisms. HD can be normalized (NHD) by dividing it by the maximum HD between corresponding vectors of all organisms. Consequently, the smaller NHD is, the more likely that *S. cerevisiae* is a good model to study the relevant process or pathways and generalize the results for the other organism. The vectors we define for each pathway include all proteins that could participate in that pathway in all organisms in the KEGG database. This ensures that the comparison we are making accounts for differences between the pathway in *S. cerevisiae* and that in the other organism and vice-versa. All calculations were performed using Mathematica [39].

## Supporting Information

Figure S1 **Frequency distribution of** ***S. cerevisiae*** **proteins according to different functional classifications**. A – Distribution according to GOSLIM biological processes. B – Distribution according to GOSLIM molecular function. C – Distribution according to GOSLIM cellular localization. D – Distribution according to KEGG pathways.
(TIF)

Figure S2 **Full heat-map representation showing how distant each organisms is from** ***S. cerevisiae*** **with respect to each individual KEGG pathway.** A green square indicates a high level of coincidence between the set of proteins involved in the specific pathway (column) in a given organism (row) and the set of proteins for the same pathway in *S. cerevisiae*. A red square indicates complete absence of the set of proteins involved in the specific pathway (column) in a given organism (row) with respect to the same pathway in *S. cerevisiae*. Intermediate colors indicate intermediate degrees of coincidence between the set of proteins in the target organism and that in *S. cerevisiae*.
(TIF)

Figure S3 **Full heat-map representation showing how distant each organisms is from** ***S. cerevisiae*** **with respect to each biological process from the GOSLIM classification.** A green square indicates a high level of coincidence between the set of proteins involved in the specific biological process (column) in a given organism (row) and the set of proteins for the same process in *S. cerevisiae*. A red square indicates complete absence of the set of proteins involved in the specific process (column) in a given organism (row) with respect to the same biological process in *S. cerevisiae*. Intermediate colors indicate intermediate degrees of coincidence between the set of proteins in the target organism and that in *S. cerevisiae*.
(TIF)

Figure S4 **Full heat-map representation showing how distant each organisms is from** ***S. cerevisiae*** **with respect to each molecular function from the GOSLIM classification.** A green square indicates a high level of coincidence between the set of proteins involved in the specific molecular function (column) in a given organism (row) and the set of proteins for the same function in *S. cerevisiae*. A red square indicates complete absence of the set of proteins involved in the specific function (column) in a given organism (row) with respect to the same molecular function in *S. cerevisiae*. Intermediate colors indicate intermediate degrees of coincidence between the set of proteins in the target organism and that in *S. cerevisiae*.
(TIF)

Figure S5 **Full heat-map representation showing how distant each organisms is from** ***S. cerevisiae*** **with respect to each cellular localization category from the GOSLIM classification.** A green square indicates a high level of coincidence between the set of proteins assigned to a specific cellular localization (column) in a given organism (row) and the set of proteins for the localization in *S. cerevisiae*. A red square indicates complete absence of the set of proteins assigned to the specific cellular localization (column) in a given organism (row) with respect to the same localization in *S. cerevisiae*. Intermediate colors indicate intermediate degrees of coincidence between the set of proteins in the target organism and that in *S. cerevisiae*.
(TIF)

Table S1 Analyzed organisms and lumped homology with respect to the *S. cerevisiae* genome.
(XLS)

Table S2 Summary of the comparison between *S. cerevisiae* sequences and those of organisms from different groups for domains, kingdoms or phyla, classified by biological process, molecular function and cellular localization from the GOSLIM ontology.
(XLS)

Table S3 Summary of the comparison between *S. cerevisiae* sequences and those of organisms from different groups for domains, kingdoms or phyla, classified with the ifferent KEGG pathways.
(XLS)

Table S4 **A comparison of dynamic and adaptive responses of different organisms with** ***S. cerevisiae*** **.** We find that organisms that are more distant to S. cerevisiae in Figures 2–5 (Figures S1–S4) with respect to some biological process also have phenotypic behavior that is more different from the yeast than those that are predicted to be closer with respect to that process.
(XLS)

Appendix S1 Appendix containing the detailed analysis of the comparison between *S. cerevisiae* and the different organisms with respect to the different KEGG pathways and GO categories.
(DOC)

Text S1 Supplementary File containing the ScCOGs.
(TXT)

Text S2 Supplementary File containing the ScCHGs.
(TXT)
