# Supplementary figures and images for: Saccharomyces cerevisiae as a Model Organism: A Comparative Study
Source: PLoS One. 2011 Feb 2;6(2):e16015. doi: 10.1371/journal.pone.0016015 (PMC3032731; doi:10.1371/journal.pone.0016015)

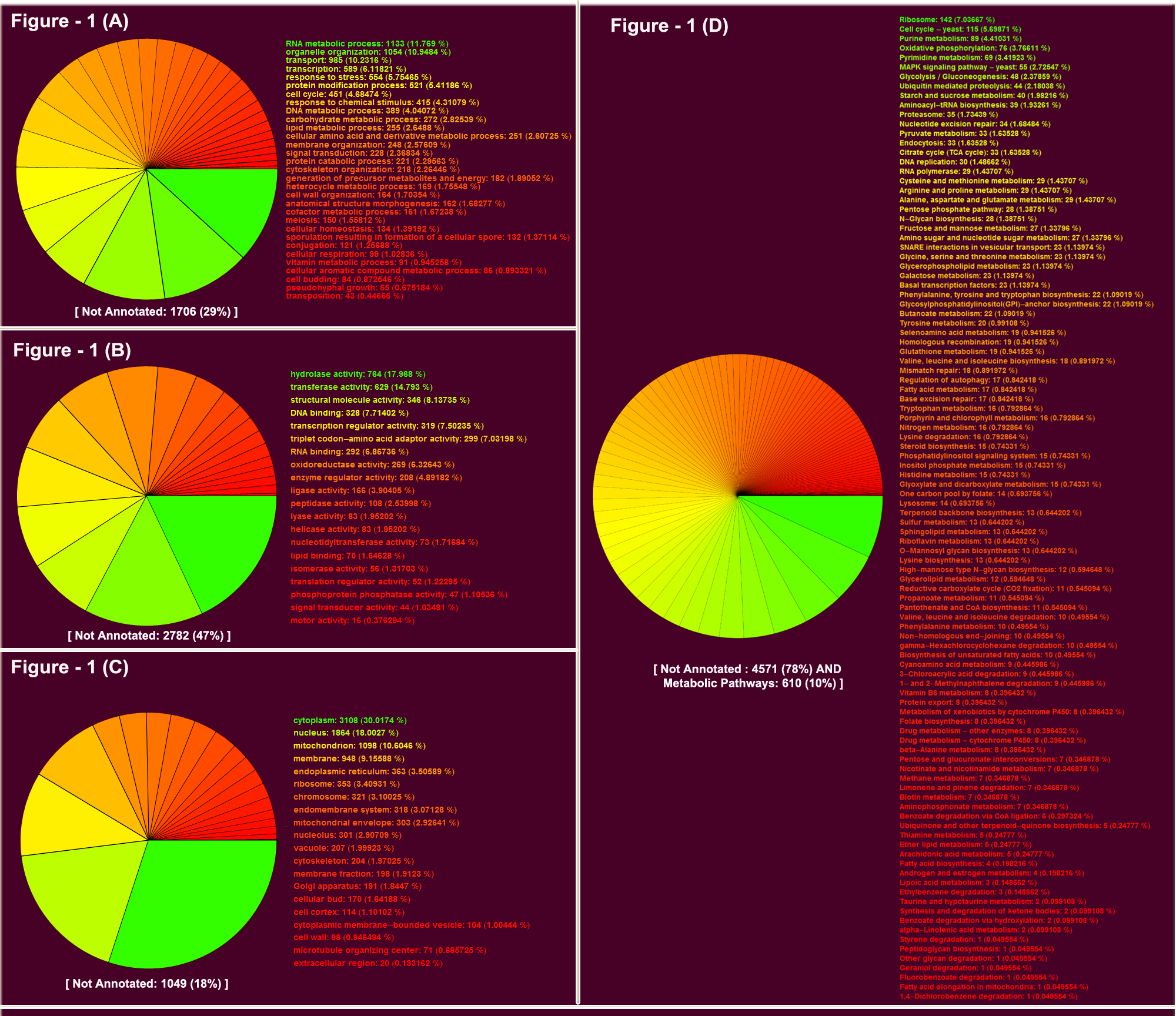

Supplement: Figure S1 — Frequency distribution of S. cerevisiae proteins according to different functional classifications. A – Distribution according to GOSLIM biological processes. B – Distribution according to GOSLIM molecular function. C – Distribution according to GOSLIM cellular localization. D – Distribution according to KEGG pathways. (TIF) [file pone.0016015.s001.tif]

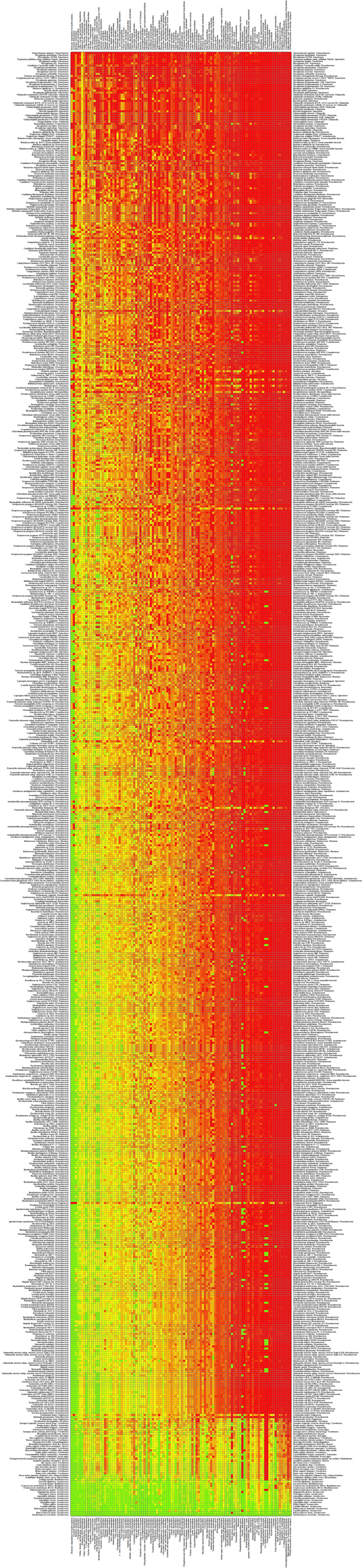

Supplement: Figure S2 — Full heat-map representation showing how distant each organisms is from S. cerevisiae with respect to each individual KEGG pathway. A green square indicates a high level of coincidence between the set of proteins involved in the specific pathway (column) in a given organism (row) and the set of proteins for the same pathway in S. cerevisiae. A red square indicates complete absence of the set of proteins involved in the specific pathway (column) in a given organism (row) with respect to the same pathway in S. cerevisiae. Intermediate colors indicate intermediate degrees of coincidence between the set of proteins in the target organism and that in S. cerevisiae. (TIF) [file pone.0016015.s002.tif]

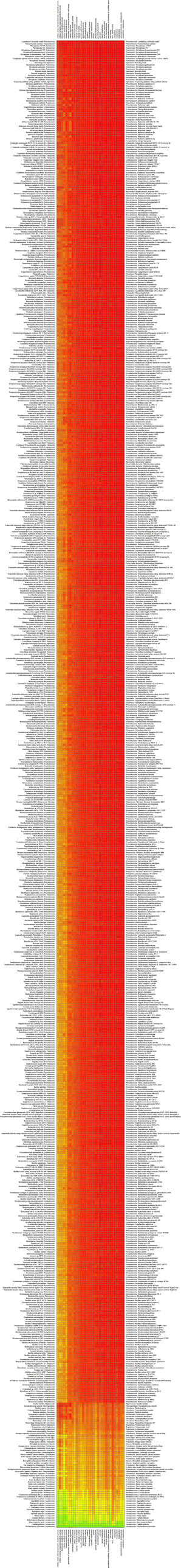

Supplement: Figure S3 — Full heat-map representation showing how distant each organisms is from S. cerevisiae with respect to each biological process from the GOSLIM classification. A green square indicates a high level of coincidence between the set of proteins involved in the specific biological process (column) in a given organism (row) and the set of proteins for the same process in S. cerevisiae. A red square indicates complete absence of the set of proteins involved in the specific process (column) in a given organism (row) with respect to the same biological process in S. cerevisiae. Intermediate colors indicate intermediate degrees of coincidence between the set of proteins in the target organism and that in S. cerevisiae. (TIF) [file pone.0016015.s003.tif]

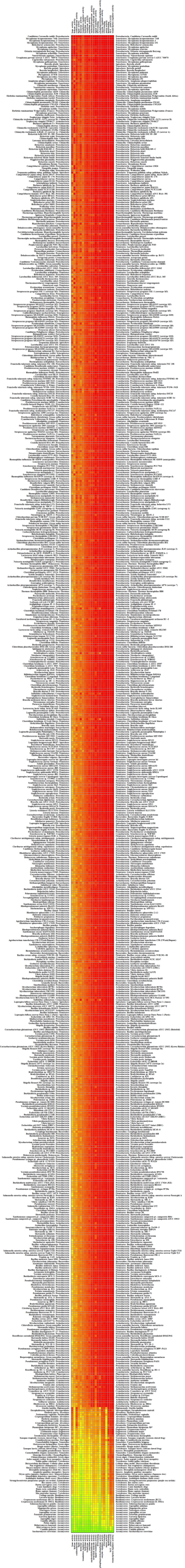

Supplement: Figure S4 — Full heat-map representation showing how distant each organisms is from S. cerevisiae with respect to each molecular function from the GOSLIM classification. A green square indicates a high level of coincidence between the set of proteins involved in the specific molecular function (column) in a given organism (row) and the set of proteins for the same function in S. cerevisiae. A red square indicates complete absence of the set of proteins involved in the specific function (column) in a given organism (row) with respect to the same molecular function in S. cerevisiae. Intermediate colors indicate intermediate degrees of coincidence between the set of proteins in the target organism and that in S. cerevisiae. (TIF) [file pone.0016015.s004.tif]

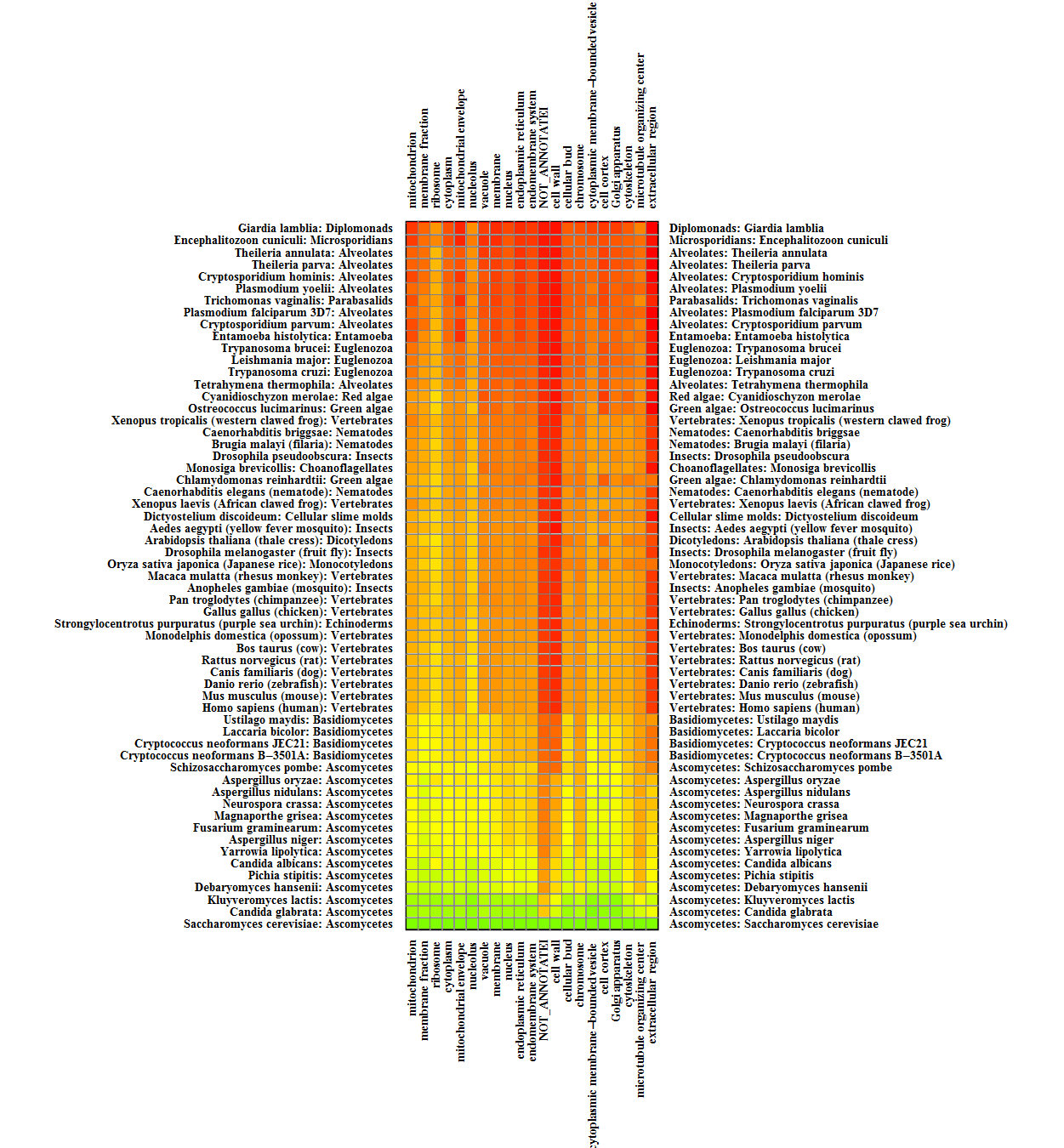

Supplement: Figure S5 — Full heat-map representation showing how distant each organisms is from S. cerevisiae with respect to each cellular localization category from the GOSLIM classification. A green square indicates a high level of coincidence between the set of proteins assigned to a specific cellular localization (column) in a given organism (row) and the set of proteins for the localization in S. cerevisiae. A red square indicates complete absence of the set of proteins assigned to the specific cellular localization (column) in a given organism (row) with respect to the same localization in S. cerevisiae. Intermediate colors indicate intermediate degrees of coincidence between the set of proteins in the target organism and that in S. cerevisiae. (TIF) [file pone.0016015.s005.tif]
