# Supplementary material for: Saccharomyces cerevisiae as a Model Organism: A Comparative Study
Source: PLoS One. 2011 Feb 2;6(2):e16015. doi: 10.1371/journal.pone.0016015 (PMC3032731; doi:10.1371/journal.pone.0016015)
Supplement: Appendix S1 — Appendix containing the detailed analysis of the comparison between S. cerevisiae and the different organisms with respect to the different KEGG pathways and GO categories. (DOC) [file pone.0016015.s010.doc]

**Supporting Materials**

**Appendix 1: Detailed functional analysis of S. cerevisiae as a model organism**

## Functional classification of the *S. cerevisiae* genome

The 5880 non-redundant genes of *S. cerevisiae* are obtained from NCBI (http://ncbi.nlm.nih.gov) database (December 2009). These genes are grouped according to four different functional classifications. Three of these describe the biological function of the proteins according to Gene Ontology (GO) categories [molecular function, biological process, and cellular localization]. The fourth category describes the KEGG pathways in which the proteins are involved.

With respect to the GO classification, 1725 (29%) proteins are not annotated, 1646 (28%) proteins are associated with a single term and 2509 (43%) proteins are associated with more than one term for biological process. Further details about the functional GO classification of *S. cerevisiae* proteins can be found in Appendix 1.

Mapping the total *S. cerevisiae* protein set to KEGG pathways terms shows that 78% (4571) of genes have not been associated with any pathway. The remaining 22% (1309) of proteins are associated to 105 terms for pathways. Almost all these proteins are associated with more than one pathway. The results are summarized in Supplementary Figure 1 and in Supplementary Tables II and III.

With this functional classification of *S. cerevisiae* proteins in place, we can compare the different molecular circuits and processes of yeast their analogs in other organisms. To do this we downloaded the fully sequenced genome of 704 organisms, distributed in three domains (Eukaryota, Bacteria and Archaea). A list of all organisms is given as Supplementary Table I.

**Functional comparison of the full *S. cerevisiae* protein complement to that of other organisms**

To compare molecular circuits, biological process, molecular function and cellular localization between *S. cerevisiae* and the other organisms, we created clusters of **orthologs** (ScCOGs), **homologues** (ScCHGs) and **absent** genes (ScCAGs) for each *S. cerevisiae* protein with respect to the genome of each of the other 704 organisms. The results are summarized in Supplementary Tables I-III and the detailed clusters can be downloaded in the supplementary files. Each cluster was associated with the functional terms corresponding to its *S. cerevisiae* protein, as classified in the previous section. Supplementary Figures 2-5 describe these results. Given the functional associations and the ScCOGs, we can now look for differences and similarities in specific pathways, functions, or processes between *S. cerevisiae* and any of the studied organisms (for more details see Supplementary Tables I-III).

**ARCHAEA DOMAIN**

We analyzed 48 species of *Archaea*. About 20% (1158) of all *S. cerevisiae* proteins generate ScCOGs that contain *Archaea* sequences. However, only 2% (103) of all yeast proteins generate ScCOGs that contain at least a sequence from each sequenced species of *Archaea*. An additional 18 (0.3%) *S. cerevisiae* proteins have homologues in all *Archaea*. 3672 (62%) *S. cerevisiae* proteins are absent in all *Archaea*. Most of these have unknown function.

At the phyla level, *Crenarchaeota* commonly share orthologs to 164 (3%) *S. cerevisiae* proteins, while *Euryarchaeota* commonly share orthologs to 148 (3%) *S. cerevisiae* proteins. *Korarchaeota* and *Nanoarchaeota* are represented in our sampling with only one organism per phylum.

Globally, the biological pathways of *S. cerevisiae* that share the highest fraction of their protein complements with all *Crenarchaeota* are “Aminoacyl-tRNA biosynthesis“ and “Proteasome“. Even so, less than 60% of the *S. cerevisiae* proteins associated with these pathways have orthologs in the organisms of the phylum. 35% of the *S. cerevisiae* proteins associated with “RNA metabolic process” have orthologs in all the organisms of the phylum. Orthologs for the protein complements of the remaining *S. cerevisiae* proteins associated with other pathways and processes are mostly absent from the phylum.

There are 3672 (62%) *S. cerevisiae* genes those are totally absent in all *Archaea*. 88% of these genes have no associated function in the KEGG database. No significant homology is found in any Archaea with respect to all of proteins from *S. cerevisiae* that are involved in “SNARE interaction vesicular transport” pathways (23 genes). Homologues for these proteins are also absent from all *Bacteria*, which is consistent with the fact that the function is very specific to eukaryotes [47]. Homologues for more than 80% of all *S. cerevisiae* proteins that are involved in “Glycosylphosphatidylinositol (GPI)-anchored biosynthesis”, “High-mannose type N-glycan biosynthesis” and “Unsaturated fatty acid biosynthesis” are absent from *Archaea*.

Homologues for 4209 (72%) *S. cerevisiae* proteins are absent in all *Crenarchaeota*, while only 3807 (65%) are absent in all *Euryarchaeota*. More than 80% of all *S. cerevisiae* proteins involved in “Ubiquitin mediated proteolysis”, “Endocytosis”, “Fructose and mannose metabolism”, “Mismatch repair”, “sphingolipid metabolism”, “High-mannose type N-glycan biosynthesis”, “Biosynthesis of unsaturated fatty acid” are absent in all *Crenarchaeota*. In contrast, all *Euryarchaeota* have significant homologues for more than 40% of the *S. cerevisiae* proteins involved in “Mismatch repair” and “Sphingolipid metabolism”. These results suggest that, for these pathways, *Euryarchaeota* are closer to *S. cerevisiae* than *Crenarchaeaota*.

**BACTERIA DOMAIN**

We analyzed 598 species of bacteria. 1612 (27%) of all *S. cerevisiae* proteins generate ScCOGs that contain bacteria sequences. However, no ScCOG or ScCHG contains a sequence from each bacterial species. Furthermore, 2881 (49%) *S. cerevisiae* genes are absent from all *Bacteria*, a smaller percentage than that for *Archaea*.

Interestingly, a higher percentage of *S. cerevisiae* proteins that participate in the “RNA polymerase”, “DNA replication”, “Pyrimidine metabolism”, and “Ribosome” pathways is absent from *Bacteria* than from *Archaea*. This suggests that, for these pathways, *S. cerevisiae* may be more similar to *Archaea* than to *Bacteria*. On the other hand, a higher percentage of genes that participate in the “Starch and sucrose metabolism”, “O-Mannosyl glycan biosynthesis”, “High-mannose type N-glycan biosynthesis”, “Biosynthesis of unsaturated fatty acid” and “Androgen and estrogen metabolism” pathways in *S. cerevisiae* is absent from *Archaea* than from *Bacteria*.

Most bacteria with fully sequenced genomes are *Proteobacteria* (315 organisms) and *Firmicutes* (122 organisms). Only 11 (0.2%) *S. cerevisiae* proteins have orthologs in all *Proteobacteria*, while 96 (2%) *S. cerevisiae* proteins have orthologs in all *Firmicutes*. One additional protein (0.01%) has homologues in all *Proteobacteria*, while an additional 8 (0.1%) proteins have homologues in all *Firmicutes*. By and large, the sets of proteins from *S. cerevisiae* that associate with individual biological processes and pathways are closer to the corresponding set of proteins from *Proteobacteria* than to those from *Firmicutes*. The exception to this rule is observed for the “Lysosome” pathway of *S. cerevisiae*, which has more absent genes in *Proteobacteria* than in *Firmicutes*.

Our dataset contains genomes for 48 *Actinobacteria* and 30 *Cyanobacteria*. *S. cerevisiae* has 180 proteins that are present in all *Actinobacteria* genomes [146 orthologs (3%) + 34 (0.6%) homologues], and 352 proteins that are present in all *Cyanobacteria* genomes [263 (4%) orthologs and 89 (2%) homologues]. The set of proteins associated with “Aminoacyl-tRNA biosynthesis”, “Pentose phosphate pathways”, “Valine, leucine and isoleucine biosynthesis”, “Histidine metabolism” pathways, “Cellular amino acid and derivative metabolic process”, “Generation of precursor metabolites and energy”, “Cofactor metabolic process” and “Cellular respiration” in *S. cerevisiae* are more similar to the corresponding sets in *Cyanobacteria* than to those in *Actinobacteria*.

3889 (66%) of all *S. cerevisiae* proteins are absent in all sequenced *Actinobacteria* and 3940 (67%) proteins of *S. cerevisiae* are absent in all *Cyanobacteria*. In terms of biological function, the sets of proteins that lack a higher number of homologues in *Actinobacteria* than in *Cyanobacteria* are associated with “Proteasome”, “Amino sugar and nucleotide sugar metabolism”, “Galactose metabolism”, “Pentose and glucuronate interconversions”, and “Lipid metabolic process”.

Other bacterial phyla have a smaller number of organisms with fully sequenced genomes [*Tenericutes* (19 organisms), *Spirochete* (11 organisms), *Bacterioides* (11 organisms), *Green nonsulfur bacteria* (8 organisms), *Chlamydia* (13 organisms), *Hyperthermophilic bacteria* (4 organisms), *Green sulfur bacteria* (7 organisms) and *Deinococcus-thermus* (4 organisms)]. The set of *S. cerevisiae* proteins that is associated with the “Aminoacyl-tRNA biosynthesis” pathway is that which is most conserved in all organisms from these phyla, with the exception of *Tenericutes*. In this phylum, only 3 genes associated to “Aminoacyl-tRNA biosynthesis” have homologues. Three out of these seven phyla have a similar number of organisms with fully sequenced genomes. Those phyla are *Spirochete*, *Bacterioides* and *Chlamydia*. The set of proteins involved in “Glycolysis/Gluconeogenesis” and “TCA cycle” pathways in *S. cerevisiae* is more similar to that of *Chlamydia* than to those of the two other phyla. The set of proteins associated with “One carbon pool by folate” in *S. cerevisiae* is more similar to that of *Bacterioides* than to those in the other phyla. All the three phyla have an equal level of similarity to *S. cerevisiae* with respect to the “Proteosome” pathway. The genes involved in “SNARE interaction in vesicular transport” pathway are totally absent in all the three phyla.

Another interesting comparison is that between *Green nonsulfur bacteria* (8 organisms) and *Green sulfur bacteria* (7 organisms). The set of *S. cerevisiae* proteins involved in “Glycolysis/Gluconeogenesis”, “Glycine, serine and threonine metabolism”, “Histidine metabolism”, “Riboflavin metabolism”, “Limonene and pinene degradation” and “Thiamine metabolism” pathways are more similar to the corresponding sets of *Green sulfur bacteria* than to those of *Green nonsulfur bacteria*. Similar fractions of the sets of *S. cerevisiae* proteins involved in “Phenylalanine, tyrosine and tryptophan biosynthesis”, “One carbon pool folate” and “Fatty acid biosynthesis” pathways are found in both phyla.

More than 80% of the *S. cerevisiae* proteins associated with “Endocytosis”, “RNA polymerase”, “Basal transcription factor”, “Glycosylphosphatidylinositol(GPI)-anchored biosynthesis”, “Porphyrin and chlorophyll metabolism”, “Steroid biosynthesis”, “Sulfur metabolism” and “High-mannose type N-glycan biosynthesis” are absent in both phyla.

**EUKARYOTA DOMAIN**

Overall, there are 59 species of eukaryotes in our dataset. About 4.5% (263) of all ScCOGs contain sequences from each of these organisms. Between 40% and 60% of all *S. cerevisiae* proteins involved in “MAPK signaling pathways”, “signal transduction” biological process, and “helicase activity” molecular functions are present in all 59 species. Furthermore, between 60% and 80% of all proteins involved in “Microtubule organizing center” of *S. cerevisiae* are also found in all 59 sequenced eukaryotes.

**FUNGI KINGDOM**

We analyze 19 fungal species. 781 (13%) of the ScCOGs contain sequences from all these species. More than 80% of the proteins of *S. cerevisiae* involved in “O-mannosyl glycan biosynthesis”, “Synthesis and degradation of ketone bodies”, “Microtubule organizing center”, “helicase activity” and “motor activity” are also present in all other *Fungi*. More than 60% of all *S. cerevisiae* proteins involved in “RNA metabolic process”, “Organelle organization”, “Protein modification process”, “Cell cycle”, “Response to stress”, “DNA metabolic process”, and “Response to chemical stimuli” are also present in all other fungi.

2310 (39%) ScCOGs contain sequences from *Basidiomycetes* (4 organisms), while only 2174 (36%) ScCOGs contain sequences from *Ascomycetes* (14 organisms) (*S. cerevisiae*’s phylum). 469 (8%) ScCHGs have sequences from all *Basidiomycetes*, while 1525 (26%) genes are absent in all sequenced *Basidiomycetes*.

Even though *S. cerevisiae* is an *Ascomycetes*, the sets of *S. cerevisiae* proteins involved in “Basal transcription factor”, “Glycerophospholipid metabolism”, “Tyrosine metabolism”, “High-mannose type N-glycan biosynthesis” and “Ether lipid metabolism” are more similar to the corresponding sets of *Basidiomycetes* than to those of other *Ascomycetes*. This is also true for the sets of proteins involved in the following biological processes: “transport”, “lipid metabolic process”, “cellular amino acid derivative metabolic process”, “membrane organization”, “generation of precursor metabolites and energy”, “heterocycle metabolic process”, “meiosis” and “Vitamin metabolic process”, molecular functions like “Structural molecular activity”, “RNA binding”, “Oxidoreductase activity”, “Nucleotidyltransferase activity” and “isomerase activity” ScCOGs sequence for cellular localization at cytoplasm, membrane and ribosomes. This suggests that for these pathways and biological processes *S. cerevisiae* might be closer to *Basidiomycetes* than to other organisms of its own phylum. The protein complements associated with the remaining pathways and biological processes in *S. cerevisiae* appear to be 80% similar to those of other *Ascomycetes.* Thus, as expected based on its evolutionary history, most *S. cerevisiae* biological processes are more similar to those of *Ascomycetes* than to those of *Basidiomycetes*.

Of all fungi, *Encephalitozoon cuniculi* is the organism with the lowest number of proteins that are similar to those of *S. cerevisiae.* Only 1764 *S. cerevisiae* proteins are also found in *E. cuniculi* [1015 (17%) orthologs and 749 (13%) homologues]. 4097 (70%) of the proteins from *S. cerevisiae* are absent from *E. cuniculi*. The sets of proteins associated with the following pathways and processes in *S. cerevisiae* are absent from *E. cuniculi*: “TCA cycle”, “Arginine and proline metabolism”, “Cysteine and methionine metabolism”, “N-glycan biosynthesis”, “SNARE interaction in vesicular transport”, “Nitrogen metabolism”, “Steroid biosynthesis”, “Sulfur metabolism”, “1- and2- Methylnaphthalene degradation”, “3-chloroacrylic acid degradation”, “Cellular amino acid and derivative metabolic process”, “Generation and precursor metabolites and energy”, “Heterocycle metabolic process”, “Cellular respiration”, “Vitamin metabolic process”, and “Cellular aromatic compound metabolic process”. Supplementary Figures 2-5 detail which other functional groups of proteins differ the most between the two organisms.

**ANIMAL KINGDOM**

We analyzed the genomes of 20 animal species, distributed throughout 4 phyla: Vertebrates (12 organisms), Insects (4 organisms), Nematodes (3 organisms), and Echinoderms (1 organism, *Strongylocentrotus purpuratus* [purple sea urchin]). 2737 (47%) ScCOGs contain animal sequences. 480 (8%) of the ScCOGs contain sequences from all animals. An additional 81 (1%) *S. cerevisiae* proteins also have homologues in all animals.

More than 60% of the *S. cerevisiae* proteins that are associated with “MAPK signaling pathways - yeast”, “Fatty acid metabolism”, “Limonene and pinene degradation pathways” are also present in all animals. Between 40% and 60% of the *S. cerevisiae* proteins associated with “Signal transduction” and between 60% and 80% of *S. cerevisiae* proteins associated with “Signal transducer activity” and “Cytoskeleton, cellular bud, and ”Microtubule organizing center” are also found in all animals.

2028 (34%) yeast proteins are absent in all the animal genomes. Most of these proteins have unknown biological function. Between 40% and 60% of the proteins involved in “Cell wall organization”, “Sporulation”, and “Transcription regulator activity” in *S. cerevisiae* are absent from all animals. This is expected, given that animals do not have cell walls.

Globally, 573 (10%) ScCOGs have sequences from all sequenced Vertebrates. This is phylum that has the lowest number of proteins that are common to all its organisms and have orthologs in *S. cerevisiae*. Homologues for the proteins from *S. cerevisiae* associated with the following processes are mostly absent from Vertebrates : “MAPK signaling pathway yeast”, “Protein modification process”, “Response to chemical stimuli”, “Signal transduction”, “Meiosis”, “Transposition”; molecular functions involved genes like “RNA binding”, “Translation regulator activity” and “Signal transducer activity”. This suggests that *S. cerevisiae* is not a good model to study these processes in vertebrates.

The sets of *S. cerevisiae* proteins involved in “Starch and sucrose metabolism”, “Galactose metabolism”, “GPI-anchored biosynthesis”, “Porphyrin and chlorophyll metabolism”, “One carbon pool by folate”, “O-Mannosyl glycan biosynthesis”, “Gamma-Hexachlorocyclohexane degradation”, “Protein modification process”, “Carbohydrate metabolic process”, “Cellular amino acid and derivative metabolic process”, “Heterocycle metabolic process” are more similar to the analogous sets found in *Insects* than to those found in *Nematodes*. The sets of *S. cerevisiae* proteins involved in “DNA metabolic process”, “Helicase activity” and “Lipid binding” are more similar to the analogous sets found in *Nematodes* than to those found in *Insects*. Both phyla have orthologs for a similar proportion of the *S. cerevisiae* proteins involved in: “Ribosomes”, “Ubiquitin mediated proteolysis”, “Aminoacyl-tRNA biosynthesis”, “TCA cycle”, “DNA replication”, “Glutathione metabolism”, “Phosphatidylinositol signaling system”, “Valine, leucine and isoleucine degradation” and “beta-Alanine metabolism”, “RNA metabolic process”, “Response to chemical stimulus”, “Transferase activity”, “DNA binding” and “Enzyme regulatory activity”. Both phyla have orthologs for a similar proportion of the *S. cerevisiae* proteins localized to: “Nucleolus”, “membrane fraction”, “Golgi apparatus”, and “Cytoplasmic membrane bounded vesicle”.

**PLANTS KINGDOM**

We analyzed 5 plant organisms distributed throughout 4 phyla. The *Dicotyledons*, *Monocotyledons*, and Red algae have one fully sequence genome each, while two Green algae genomes have been fully sequenced. 1371 (23%) ScCOGs contain sequences from all plants. An additional 253 (4%) yeast proteins have homologues in all plants.

All plants have orthologs for more than 80% of the *S. cerevisiae* proteins associated with: ”Ribosome”, “Aminoacyl-tRNA biosynthesis”, “Pentothenate and CoA biosynthesis”, “Propeonate metabolism”, “Valine, leucine and isoleucine degradation”, “Limonene and pinene degradation”, “Homologous recombination”, “Selenoamino acid metabolism”, “Mismatch repair”, “Valine, leucine and isoleucine biosynthesis”, “Lysosome” ,“Alpha-Linolenic acid metabolism”, “Benzoate degradation via hydroxylation”, “Meiosis”, “Structure molecular activity”, “Ligase activity”, “Helicase activity”, and ”Isomerase activity”. All plants have orthologs for more than 80% of the set of *S. cerevisiae* proteins localized at “Ribosomes”.

Interestingly, in Dicotyledons only 282 (5%) *S. cerevisiae* proteins are absent, whereas in *Monocotyledons* and *Red algae* at least 43% of *S. cerevisiae* proteins are absent. It is not possible at this time to know if this difference is just a consequence of the very limited sampling of plant genomes that is available for our analysis or if it reflects some fundamental difference between the phyla. Nevertheless, *Arabidopsis thaliana* has the protein complement that is closest to that of *S. cerevisiae* in the plant kingdom.

**PROTISTS KINGDOM**

We analyzed 15 protists organisms distributed throughout 7 phyla. Cellular slime molds, *Choanoflagellates*, *Diplomonads*, *Entamoeba* and *Parabasalids* have only one fully sequenced genome each, while *Alveolates* has 7 and *Euglenozoa* has 3 fully sequenced genomes. 591 (10%) ScCOGs contain sequences from all protists. An additional 43 (0.7%) yeast proteins have homologues in all protists. Between 60% and 80% of all *S. cerevisiae* proteins associated with the “Proteasome” have orthologs in all sequenced organisms from the Protists kingdom. Between 40% and 60% of the proteins associated with the following pathways in *S. cerevisiae* have orthologs in all protists: “Ribosome”, “MAPK signaling pathway - yeast”, “Ubiquitin mediated proteolysis”, “Aminoacyl-tRNA biosynthesis”, “Nucleotide excision repair”, “Endocytosis”, “DNA replication”, “Homologous recombination”, “Mismatch repair”, “Lysosome”, and “Protein export”.

839 (14%) *S. cerevisiae* proteins have orthologs and 194 (3%) *S. cerevisiae* proteins have homologues in *Alveolates*. Orthologs or homologues for more than 80% of the *S. cerevisiae* proteins involved in “Proteosome” pathways are also presents in Alveolates. Between 60% and 80% of the proteins involved in the following pathways of *S. cerevisiae* have orthologs and/or homologues in Alveolates: “Ribosome”, “Aminoacyl-tRNA biosynthesis”, “Pyrimidine metabolism”, “MAPK signaling pathway - yeast”, “Ubiquitin mediated proteolysis”, “Nucleotide excision repair”, “DNA replication”, “Homologous recombination”, “Mismatch repair“, “Aminoacyl-tRNA biosynthesis”, “Fatty acid metabolism”, “Nitrogen metabolism”, “Glyoxylate and dicarboxylate metabolism”, and “CO2 fixation”.

2740 (47%) *S. cerevisiae* proteins are absent in *Alveolates*. Homologues and orthologs for the *S. cerevisiae* proteins involved in the following pathways are absent from the genome of all sequenced *Alveolates*: “O-Mannosyl glycan biosynthesis”, “Riboflavin metabolism” and “High-mannose type N-glycan biosynthesis”.

In *Euglenozoa*, 1281 (22%) *S. cerevisiae* proteins have orthologs and 695 (12%) *S. cerevisiae* proteins have homologous. Orthologs and homologues for more than 80% of the *S. cerevisiae* proteins involved in the following pathways are also found in *Euglenozoa*: “Ribosome”, “Proteosome”, “Citrate cycle (TCA cycle)”, “Glutathione metabolism”, “Homologous recombination”, “Mismatch repair”, “Inositol phosphate metabolism”, “Phosphatidylinositol signaling system”, “Lysosome”.

3157 (54%) *S. cerevisiae* proteins are absent in *Euglenozoa*. Most of these are also absent in *Alveolates*.

**Proteins that are specific to *S. cerevisiae***

There are 24 *S. cerevisiae* proteins that have no orthologs in any other organism. However, out of these, only ten have no homologues in any of the analyzed genomes. The NCBI references for these proteins are NP_010097, NP_010148 (ribosomal protein L47 of 60S subunit), NP_010496, NP_013364, NP_878067, NP_010319, NP_013978, NP_878042, NP_878075, NP_878108. These ten genes code for small peptides. A few of them may be miss-annotated as genes. However, some have been predicted based on microarray expression data, which strongly suggests that they are being expressed and may have a function that is specific to this yeast.

**Functional comparison of biological processes and pathways between *S. cerevisiae* and other organisms**

**KEGG Pathways**

Figure 1 summarizes the results for KEGG pathways (for more detail analysis see Supplementary Figure 2). Here, we find that “Benzoate degradation via hydroxylation” (2 genes) is the biological pathway that is fully present in the highest fraction of organisms. Even so, this pathway is fully absent from all *Tenericutes organisms*. “Geraniol degradation” (1 gene), “Methane metabolism” (7 genes), “Propanoate metabolism” (11 genes), “Valine, leucine and isoleucine degradation” (18 genes), “Aminoacyl-t-RNA biosynthesis” (39 genes) and “Glycolysis/Gluconeogenesis” (48 genes) are also pathways that appear to be similar to those of *S. cerevisiae* in a large fraction of organisms. Pathways such as *S. cerevisiae*’s “RNA polymerase” (29 genes), “Lysosome” (14 genes), “Endocytosis” (33 genes), “Oxidative phosphorylation” (76 genes), “Ribosome” (142 genes), “MAPK signaling pathway - yeast” (55 genes), “DNA replication” (30 genes), and “Ubiquitin mediated proteolysis” (44 genes) and “Nucleotide excision repair” (34 genes) are much more similar to those from other eukaryotes than to the corresponding prokaryotic pathways (when they exist).

The full “Valine, leucine and isoleucine” pathway (18 genes) is found in all eukaryotes. Of all pathways, this is the one that is closest to that of a largest fraction of *Proteobacteria*, *Actinobacteria* and *Firmicutes*. Other prokaryotic phyla only have orthologs for less than 40% of the proteins in the pathway.

The *S. cerevisiae* “DNA replication” pathway (30 genes) is similar to that of all other eukaryotes and *Archaea*. *Bacteria* have no orthologs to protein associated with the yeast pathway. However, sequence homologues for the pathway are present in the *Bacteria* domain. *S. cerevisiae* “MAPK signaling pathways” (55 genes) are also well conserved in *Fungi*, and partially conserved in Animals, Plants and Protists. The *S. cerevisiae* “Ubiquitin mediated proteolysis” pathway (44 genes) is similar to those of other *Fungi*, *Animal*, *Plants,* and *Protists*. Orthologs for proteins involved in this pathway are often absent in *Alveolates*. The “Proteasome” pathway (35 genes) is similar to that of most *Eukaryotes*, with *Diplomonads* being the exception. These organisms have orthologs for only a few genes of the pathway.

The “Glycolysis/Gluconeogenesis” pathway (48 genes) is very similar between *S. cerevisiae* and all *Eukaryotes* and most *Bacteria* and *Archaea*, although, all the *Proteobacteria* being the exception. The *S. cerevisiae* “Thiamine metabolism” pathway (5 genes) is most similar to the corresponding pathways in other *Fungi*, in *Plants*, and in some *Proteobacteria*. The pathway is absent in *Animals* and *Protists*. The “Steroid biosynthesis” pathway (15 genes) is fully present in *Fungi, Plants* and *Vertebrates*. In *Insects* and *Nematodes* the pathway is absent.

The *S. cerevisiae* “Basal transcription factor” (23 genes) and “High-mannose type N-glycan biosynthesis” (12 genes) are similar only to the corresponding pathways of other *Ascomycetes*. Nevertheless, a fraction of the proteins for the first pathway are present in human, dog, zebra fish and African clawed frog. The “SNARE interaction in vesicular transport” pathway (23 genes) is fully present only in *Kluyveromyces lactis, Candida glabrata* and *Pichia stipitis*. It is completely absent from other *Ascomycetes*, from *Basidiomycetes*, from *Animals*, and from *Plants*.

The pathway that executes *S. cerevisiae* cell cycle (115 genes) appears to be quite unique to Fungi, because only a small fraction of its proteins have orthologs in other eukaryotes. This suggests that extrapolating the results of studying cell cycle in *S. cerevisiae* to other organisms should be done only at the level of basic principles, if at all [see for example [37, 38].

As expected, the closest organisms to *S. cerevisiae* in our analysis are *Kluyveromyces lactis* and *Candida glabrata*. *A. thaliana* (Dicotyledons) and *Oryza sativa* (Monocotyledons) are the closest organisms to *S. cerevisiae,* outside of the *Fungi* clade. A curious observation is that, when clustering organisms with respect to *S. cerevisiae*, most of the mammals remain close to *S. cerevisiae*. Humans, dogs, mice, cows and rats are among the organisms that are closer to the yeast, when you disregard other fungi. *Dictyostelium discoideum* (Cellular slime molds) is the closest protist to *S. cerevisiae*, whereas, *Giardia lamblia* (Diplomonads) is the most distant protist. Interestingly, *E. cuniculi* (*Microsporidians*) is the eukaryotic organism that appears to be the most different from *S. cerevisiae*, even though it belongs to the *Fungi* kingdom. Only some proteins from a few of the pathways from *S. cerevisiae* have orthologs in *E. cuniculi*. These pathways are “Arachidonic acid metabolism”, “Alpha linolenic acid metabolism”, “Pentose & glucuronate interconversion”, “Terpenoid backbone biosynthesis”, “Mismatch repair”, “Proteasome”, “RNA polymerase” and “Base excision repair”. This organism has what appears to be a vestigial mitochondrial organelle, the mitosome. Fe-S cluster biogenesis, which takes place in the *S. cerevisiae* mitochondria, is also initiated in the mitosome of *E. cuniculi*. The remaining *S. cerevisiae* pathways are absent in *E. cuniculi*. This is consistent with the evolutionary history of *Microsporideans* [39].

When it comes to human metabolism, *S. cerevisiae* is likely to be a reasonable model for the study of “mismatch repair” (18 genes), “Ubiquitin and other terpenoid-quinone biosynthesis” (5 genes), “Inositol phosphate metabolism” (15 genes), “Steroid biosynthesis” (15 genes), “Ubiquitin mediated proteolysis” (44 genes), “DNA replication” (30 genes), “Ribosome” (142 genes), “Proteasome” (35 genes), “Mismatch repair” (18 genes), “Galactose metabolism” (23 genes), “One carbon pool by folate” (14 genes) and “Glycolysis/gluconeogenesis” (48 genes). It might also be a moderately good model to study “basal transcription factor” pathways (17 genes), “N-Glycan biosynthesis” (28 genes), “RNA polymerase” (29 genes), “Glycine, serine and threonine metabolism” (30 genes) and “Glycerophospholipid metabolism” (16 genes). *S. cerevisiae* pathways that have orthologs in humans for only a small fraction of their proteins are: “androgen estrogen metabolism” (4 genes), “Cyanoamino acid metabolism” (9 genes), “Nitrogen metabolism” (16 genes), “SNARE interactions in vesicular transport” (23 genes), “Gamma-Hexachlorocyclohexane degradation” (10 genes), “Phenylalanine, tyrosine and tryptophan biosynthesis” (22 genes), “GPI-anchored biosynthesis” (22 genes) and “MAPK signaling pathway - yeast” (55 genes).

Most *Bacteria* are closer to *S. cerevisiae* than any *Archaea*. Specifically, *Proteobacteria* are the closest to *S. cerevisiae* and *Klebsiella pneumoniae* is the closest Proteobacteria. *Tenericutes* are the most distant bacterial phylum to *S. cerevisiae*, and *Mycoplasma genitalium* is the most distant organism. In *Archaea,* *Haloarcula marismortui* (*Euryarchaeota*) is the closest organism to *S. cerevisiae* and *Nanoarchaeum equitans* (*Nanoarchaeota*) is the most distant.

**GO Biological Processes, Cellular Component and Molecular Function**

Figures 2, 3, and 4 summarize the results for the comparisons between *S. cerevisiae* and the other organisms using the GO categories classification. The results are quite similar to those described for Figure 1, which suggests that these functional classifications could be equivalent to a large extent, in spite of all problems that they might have (see discussion). *S. cerevisiae* metabolic activities like “Cellular amino acid and derivative metabolic process”, “Cellular aromatic compound metabolic process”, “Heterocycle metabolic process”, “Cofactor metabolic process” and “Vitamin metabolic process” are the ones that are more conserved in all organisms. In contrast, “Motor activity”, “Transcription”, “Anatomical structure morphogenesis”, “Transposition”, “Conjugation”, “Cell budding”, and “Protein modification process” appear to be conserved mostly in eukaryotes. Conservation of “Cell wall organization” pathways is restricted to fungi.

## Evolutionary aspects of this work

As one would predict beforehand, the organisms that have the highest fraction of processes associated to proteins sets that are similar to the corresponding proteins sets of *S. cerevisiae* are *Kluyveromyces lactis,* *Candida glabrata*, and other Ascomycetes. *A. thaliana* (Dicotyledons) and *Oryza sativa* (Monocotyledons) are the organisms with the largest fraction of processes with protein sets that are similar to those of *S. cerevisiae,* outside of the *Fungi* clade. In general, ranking the organisms with respect to the global similarity between their protein sets and the corresponding set in *S. cerevisiae* creates a clustering tree that mostly replicates phylogenetic trees built using ribosomal RNA (data not shown).

Interestingly, in that clustering tree, *Encephalitozoon cuniculi* (*Microsporidians*) is the eukaryotic organism that is the most distant from *S. cerevisiae*, even though it belongs to the *Fungi* kingdom. Only some proteins from a few of the pathways of *S. cerevisiae* have orthologs in *E. cuniculi* (see supplementary appendix for details). This is consistent with the evolutionary history of *Microsporideans* as a specialized intracellular fungi that both, lost many of its biological functions and has a high rate of divergence from other eukaryotes [25].

Another interesting fact is that 138 (111 orthologs and 27 homologues) out of 352 *S. cerevisiae* proteins that have homologues in all *Cyanobacteria* are mitochondrial proteins. 39% of all *S. cerevisiae* genes with orthologs in all *Cyanobacteria* are mitochondrial. In contrast 13.5% of all *S. cerevisiae* genes are mitochondrial. Thus, there are 2.9 (±0.24) times more mitochondrial genes in the *Cyanobacteria* ortholog set than one would expect from change alone. Given that a) the mitochondrial ancestor is an α-proteobacteria from the *Rickettsia* genus and not a *Cyanobacteria*, and b) the ancestor of chloroplasts is a *Cyanobacteria*, this result puzzled us and we speculated that it could provide functional insight into the evolution of both organelles [26-30].

To understand if there were any genes with specific functions and strong homologues that were common to mitochondria and chloroplasts, we decided to compare the sets of genes that have strong homologues between *S. cerevisiae*, all *Cyanobacteria*, all *Rickettsia* and the plant *A. thaliana*. We discovered that, 98 out of the 111 *S. cerevisiae* mitochondrial genes that had orthologs in all *Cyanobacteria* also had orthologs localized to the chloroplast in *A. thaliana*. Out of these, 92 where predicted to have strong homologues both in mitochondria and chloroplast. The localization of genes in *A. thaliana* was determined by checking the GO annotation (cellular component) of the genes in the TAIR database [31]. We also found that 110 *S. cerevisiae* mitochondrial genes had orthologs in all *Rickettsia*. Out of these, 68 had orthologs also in all *Cyanobacteria* and in *A. thaliana*. Mitochondrial and/or chloroplast genes are 3 to 4 times more common in this data set than one would expect from the set of *S. cerevisiae* proteins. The biological processes that dominate these sets of genes according to the GO classification is “biological process unknown”. Furthermore, genes involved in energy production are also abundant. However, no specific biological process or molecular function was significantly enriched in these datasets when compared to all *A. thaliana* chloroplast and/or mitochondrial genes. Thus, further work that requires better functional classifications is needed in order to understand if these datasets have any functional implications in the evolution of energy producing organelles.
